# Ten simple rules for managing laboratory information

**DOI:** 10.1371/journal.pcbi.1011652

**Published:** 2023-12-07

**Authors:** Casey-Tyler Berezin, Luis U. Aguilera, Sonja Billerbeck, Philip E. Bourne, Douglas Densmore, Paul Freemont, Thomas E. Gorochowski, Sarah I. Hernandez, Nathan J. Hillson, Connor R. King, Michael Köpke, Shuyi Ma, Katie M. Miller, Tae Seok Moon, Jason H. Moore, Brian Munsky, Chris J. Myers, Dequina A. Nicholas, Samuel J. Peccoud, Wen Zhou, Jean Peccoud

**Affiliations:** 1 Department of Chemical and Biological Engineering, Colorado State University, Fort Collins, Colorado, United States of America; 2 Molecular Microbiology Unit, Faculty of Science and Engineering, University of Groningen, Groningen, the Netherlands; 3 School of Data Science, University of Virginia, Charlottesville, Virginia, United States of America; 4 Department of Biomedical Engineering, University of Virginia, Charlottesville, Virginia, United States of America; 5 College of Engineering, Boston University, Boston, Massachusetts, United States of America; 6 Department of Infectious Disease, Imperial College, London, United Kingdom; 7 School of Biological Sciences, University of Bristol, Bristol, United Kingdom; 8 BrisEngBio, University of Bristol, Bristol, United Kingdom; 9 Biological Systems and Engineering Division, Lawrence Berkeley National Laboratory, Berkeley, California, United States of America; 10 US Department of Energy Agile BioFoundry, Emeryville, California, United States of America; 11 US Department of Energy Joint BioEnergy Institute, Emeryville, California, United States of America; 12 LanzaTech, Skokie, Illinois, United States of America; 13 Center for Global Infectious Disease Research, Seattle Children’s Hospital, University of Washington Medicine, Seattle, Washington, United States of America; 14 Department of Energy, Environmental & Chemical Engineering, Washington University in St. Louis, St. Louis, Missouri, United States of America; 15 Department of Computational Biomedicine, Cedars-Sinai Medical Center, Los Angeles, California, United States of America; 16 Department of Electrical, Computer & Energy Engineering, University of Colorado Boulder, Boulder, Colorado, United States of America; 17 Department of Molecular Biology & Biochemistry, University of California Irvine, Irvine, California, United States of America; 18 Department of Electrical and Computer Engineering, Colorado State University, Fort Collins, Colorado, United States of America; 19 Department of Statistics, Colorado State University, Fort Collins, Colorado, United States of America; Dassault Systemes BIOVIA, UNITED STATES

## Abstract

Information is the cornerstone of research, from experimental (meta)data and computational processes to complex inventories of reagents and equipment. These 10 simple rules discuss best practices for leveraging laboratory information management systems to transform this large information load into useful scientific findings.

## Introduction

**Fig 1:**
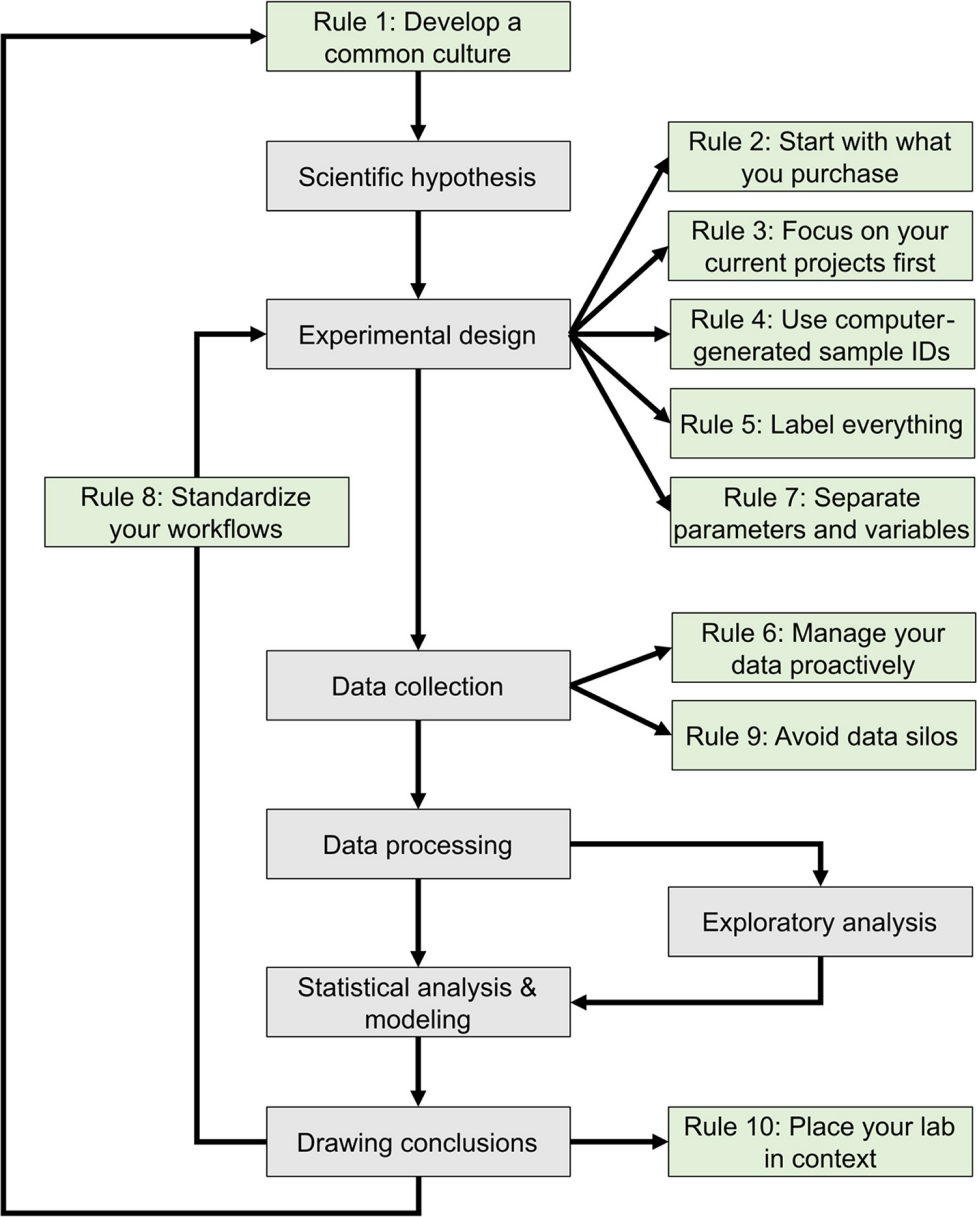
Information management enhances the experimental and modeling cycle. Our 10 simple rules for managing laboratory information (green) augment the cycle of hypothesis formulation, design, data analysis, modeling, and decision-making (gray). The experimental design phase is improved by carefully tracking your inventory, samples, parameters, and variables. Proactive data management and the thoughtful use of databases facilitate statistical and exploratory analyses as well as the development of conclusions that inform the next round of experiments. Frequent reevaluation of project, team, and workflow success is a critical component of refining experimental processes, developing a common culture, and positioning your research group in the greater scientific context.

The development of mathematical models that can predict the properties of biological systems is the holy grail of computational biology [1,2]. Such models can be used to test biological hypotheses [3], quantify the risk of developing diseases [3], guide the development of biomanufactured products [4], engineer new systems meeting user-defined specifications, and much more [4,5]. Irrespective of a model’s application and the conceptual framework used to build it, the modeling process proceeds through a common iterative workflow. A model is first evaluated by fitting its parameters such that its behavior matches experimental data. Models that fit previous observations are then further validated by comparing the model predictions with the results of new observations that are outside the scope of the initial data set (**Fig 1**).

Historically, the collection of experimental data and the development of mathematical models were performed by different scientific communities [6]. Computational biologists had little control over the nature and quality of the data they could access. With the emergence of systems biology and synthetic biology, the boundary between experimental and computational biology has become increasingly blurred [6]. Many labs and junior scientists now have expertise in both producing and analyzing large volumes of digital data produced by high-throughput workflows and an ever-expanding collection of digital instruments [7]. In this context, it is critically important to properly organize the exponentially growing volumes of experimental data to ensure they can support the development of models that can guide the next round of experiments [8].

We are a group of scientists representing a broad range of scientific specialties, from clinical research to industrial biotechnology. Collectively, we have expertise in experimental biology, data science, and mathematical modeling. Some of us work in academia, while others work in industry. We have all faced the challenges of keeping track of laboratory operations to produce high-quality data suitable for analysis. We have experience using a variety of tools, including spreadsheets, open-source software, homegrown databases, and commercial solutions to manage our data. Irreproducible experiments, projects that failed to meet their goals, datasets we collected but never managed to analyze, and freezers full of unusable samples have taught us the hard way lessons that have led to these 10 simple rules for managing laboratory information.

This journal has published several sets of rules regarding best practices in overall research design [9,10], as well as the computational parts of research workflows, including data management [11–13] and software development practices [14–16]. The purpose of these 10 rules (**Fig 1**) is to guide the development and configuration of lab information management systems (LIMS). LIMS typically offer lab notebook, inventory, workflow planning, and data management features, allowing users to connect data production and data analysis to ensure that useful information can be extracted from experimental data and increase reproducibility [17,18]. These rules can also be used to develop training programs and lab management policies. Although we all agree that applying these rules increases the value of the data we produce in our laboratories, we also acknowledge that enforcing them is challenging. It relies on the successful integration of effective software tools, training programs, lab management policies, and the will to abide by these policies. Each lab must find the most effective way to adopt these rules to suit their unique environment.

## Rule 1: Develop a common culture

Data-driven research projects generally require contributions from multiple stakeholders with complementary expertise. The project’s success depends on the entire team developing a common vision of the project objectives and the approaches to be used [19–21]. Interdisciplinary teams, in particular, must establish a common language as well as mutual expectations for experimental and publication timelines [19]. Unless the team develops a common culture, one stakeholder group can drive the project and impose its vision on the other groups. Although interdisciplinary (i.e., wet-lab and computational) training is becoming more common in academia, it is not unusual for experimentalists to regard data analysis as a technique they can acquire simply by hiring a student with computer programming skills. In a corporate environment, research informatics is often part of the information technology group whose mission is to support scientists who drive the research agenda. In both situations, the research agenda is driven by stakeholders who are unlikely to produce the most usable datasets because they lack sufficient understanding of data modeling [20]. Perhaps less frequently, there is also the situation where the research agenda is driven by people with expertise in data analysis. Because they may not appreciate the subtleties of experimental methods, they may find it difficult to engage experimentalists in collaborations aimed at testing their models [20]. Alternatively, their research may be limited to the analysis of disparate sets of previously published datasets [19]. Thus, interdisciplinary collaboration is key to maximizing the insights you gain from your data.

The development of a common culture, within a single laboratory or across interdisciplinary research teams, must begin with a thorough onboarding process for each member regarding general lab procedures, research goals, and individual responsibilities and expectations [21,22]. Implementing a LIMS requires perseverance by users, thus a major determinant of the success of a LIMS is whether end-users are involved in the development process [17,23]. When the input and suggestions of end-users are considered, they are more likely to engage with and upkeep the LIMS on a daily basis [23]. The long-term success of research endeavors then requires continued training and reevaluation of project goals and success [19,21] (**Fig 1**).

These 10 simple rules apply to transdisciplinary teams that have developed a common culture allowing experimentalists to gain a basic understanding of the modeling process and modelers to have some familiarity with the experimental processes generating the data they will analyze [19]. Teams that lack a common vision of data-driven research are encouraged to work toward acquiring this common vision through frequent communication and mutual goal setting [19,20]. Discussing these 10 simple rules in group meetings may aid in initiating this process.

## Rule 2: Start with what you purchase

All the data produced by your lab are derived from things you have purchased, including supplies (consumables), equipment, and contract manufactured reagents, such as oligonucleotides or synthetic genes. In many cases, (meta)data on items in your inventory may be just as important as experimentally derived data, and as such, should be managed according to the Findability, Accessibility, Interoperability, and Reuse (FAIR) principles for (meta)data management (https://www.go-fair.org/fair-principles/) [24]. Assembling an inventory of supplies and equipment with their associated locations can be handled in a few weeks by junior personnel without major interruption of laboratory operations, although establishing a thorough inventory may be more difficult and time-consuming for smaller labs with fewer members. Nevertheless, managing your lab inventory provides an immediate return on investment by positively impacting laboratory operations in several ways. People can quickly find the supplies and equipment they need to work, supplies are ordered with appropriate advance notice to minimize work stoppage, and data variation is reduced due to standardized supplies and the ability to track lot numbers easily [17,25,26] (**Fig 1**).

Many labs still use Excel to keep track of inventory despite the existence of several more sophisticated databases and LIMS (e.g., Benchling, Quartzy, GenoFAB, LabWare, LabVantage, TeselaGen) [25]. These can facilitate real-time inventory tracking unlike a static document, increasing the Findability and Accessibility of inventory data. While some systems are specialized for certain types of inventories (e.g., animal colonies or frozen reagents), others are capable of tracking any type of reagent or item imaginable [25]. When considering what items to keep track of, there are 3 main considerations: expiration, maintenance, and ease of access.

Most labs manage their supplies through periodic cleanups of the lab, during which they sort through freezers, chemical cabinets, and other storage areas; review their contents; and dispose of supplies that are past their expiration date or are no longer useful. By actively tracking expiration dates and reagent use in a LIMS, you can decrease the frequency of such cleanups since the LIMS will alert users when expiration dates are approaching or when supplies are running low. This can prevent costly items from being wasted because they are expired or forgotten, and furthermore, the cost of products can be tracked and used to inform which experiments are performed.

LIMS can also support the use and service of key laboratory equipment. User manuals, service dates, warranties, and other identifying information can be attached directly to the equipment record, which allows for timely service and maintenance of the equipment. Adding equipment to the inventory can also prevent accidental losses in shared spaces where it is easy for people to borrow equipment without returning it. The label attached to the equipment (Rule 5) acts as an indication of ownership that limits the risk of ownership confusion when almost identical pieces of equipment are owned by neighboring laboratories. As the laboratory inventory should focus on larger, more expensive equipment and supplies, inexpensive and easily obtained equipment (i.e., office supplies) may not need to be inventoried. An additional benefit of inventory management in a LIMS is the ability to create a record connecting specific equipment and supplies to specific people and projects, which can be used to detect potential sources of technical bias and variability (Rules 4 and 5).

## Rule 3: Focus on your current projects first

After establishing an inventory of supplies and equipment, it is natural to consider using a similar approach with the samples that have accumulated over the years in freezers or other storage locations. This can be overwhelming because the number of samples will be orders of magnitude larger than the number of supplies. In addition, documenting them is likely to require more effort than simply retrieving a product documentation from a vendor’s catalog.

Allocating limited resources to making an inventory of samples generated by past projects may not benefit current and future projects. A more practical approach is to prioritize tracking samples generated by ongoing projects and document samples generated by past projects on an as-needed basis.

### Inventory your samples before you generate them

It is a common mistake to create sample records well after they were produced in the lab. The risks of this retroactive approach to recordkeeping include information loss, as well as selective recordkeeping, in which only some samples are deemed important enough to document while most temporary samples are not, even though they may provide critical information.

A more proactive approach avoids these pitfalls. When somebody walks into a lab to start an experiment, the samples that will be generated by this experiment should be known. It is possible to create the computer records corresponding to these samples before initiating the laboratory processes that generates the physical samples. The creation of a sample record can therefore be seen as part of the experiment planning process (**Fig 1**). This makes it possible to preemptively print labels that will be used to track samples used at different stages of the process (Rule 5).

It may also be useful to assign statuses to samples as they progress through different stages of their life cycle, such as “to do,” “in progress,” “completed,” or “canceled,” to differentiate active works in progress from the backlog and samples generated by previous experiments. As the experimental process moves forward, data can be continually appended to the sample computer record. For example, the field to capture the concentration of a solution would be filled after the solution has been prepared. Thus, the success, or failure, of experiments can be easily documented and used to inform the next round of experiments.

### Develop sample retention policies

It is always unpleasant to have to throw away poorly documented samples. The best strategy to avoid this outcome is to develop policies to discard only samples that will not be used in the future, a process rendered more objective and straightforward with adequate documentation. Properly structured workflows (Rule 8) should define criteria for which samples should be kept and for how long. All lab members should be trained in these policies to ensure consistency, and policies should be revisited as new research operating procedures are initiated.

It can be tempting to keep every tube or plate that still contains something as a backup. This conservative strategy generates clutter, confusion, and reproducibility issues, especially in the absence of documentation. While it makes sense to keep some intermediates during the execution of a complex experimental workflow, the successful completion of the experiment should trigger the elimination of intermediates that have lost their purpose, have limited shelf life, and/or are not reusable. During this intermediate step, samples that are deemed as critical backup should be stored in a separate location from the working sample to minimize the likelihood of loss of both samples in case of electrical failure, fire, etc. Using clear labels (Rules 4 and 5) and storing intermediate samples in dedicated storage locations can help with the enforcement of sample disposal policies.

## Rule 4: Use computer-generated sample identification numbers

Generating sample names is probably not the best use of scientists’ creativity. Many labs still rely on manually generated sample names that may look something like “JP PCR 8/23 4.” Manually generated sample names are time-consuming to generate, difficult to interpret, and often contain insufficient information. Therefore, they should not be the primary identifier used to track samples.

Instead, computer-generated sample identification numbers (Sample ID) should be utilized as the primary ID as they are able to overcome these limitations. Rather than describing the sample, a computer-generated sample ID provides a link between a physical sample and a database entry that contains more information associated with the sample. The Sample ID is the only piece of information that needs to be printed on the sample label (Rule 5) because it allows researchers to retrieve all the sample information from a database. A sample tracking system should rely on both computer-readable and human-readable Sample IDs.

### Computer-readable IDs

**Fig 2:**
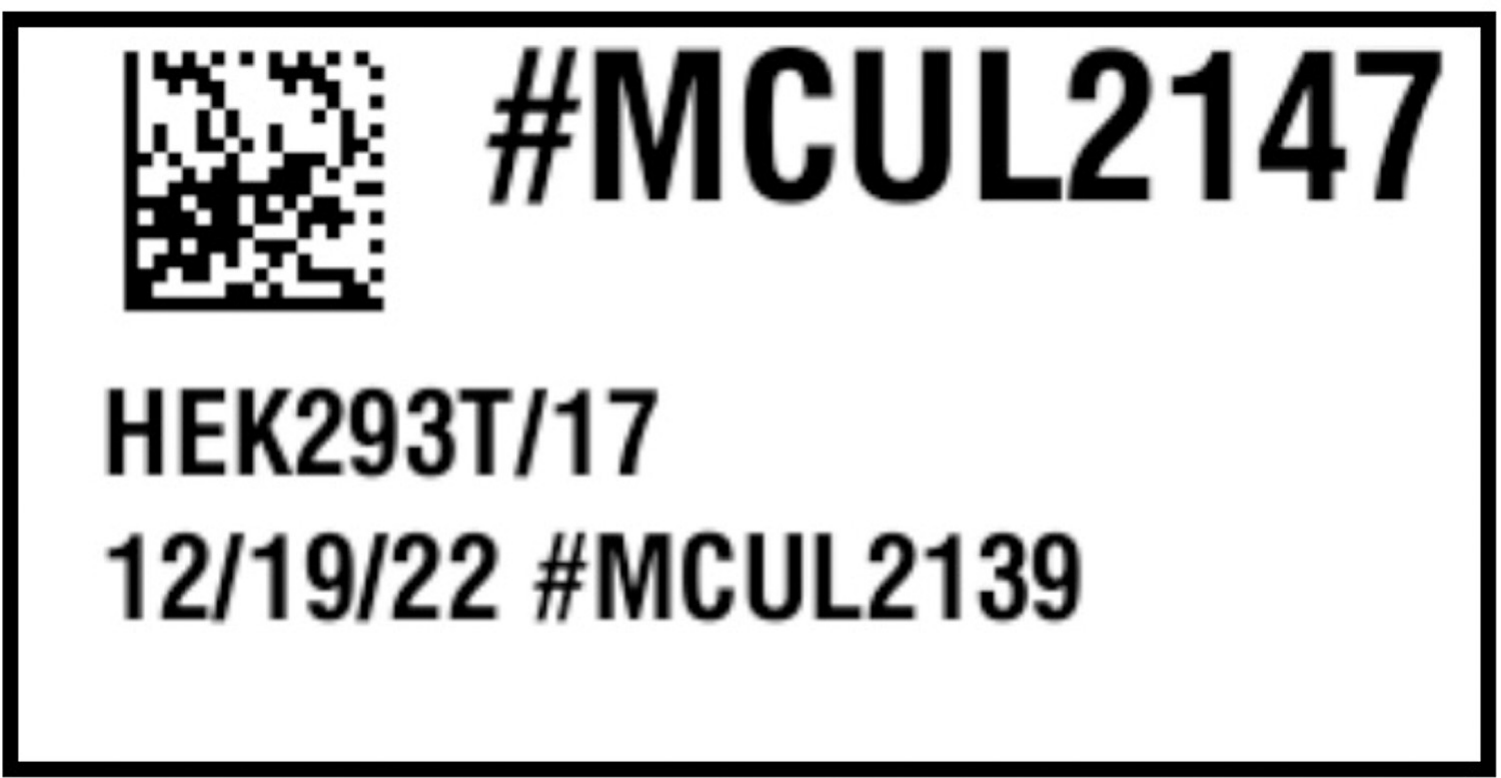
Sample label. The first line includes a unique computer-readable barcode as well as a human-readable computer-generated sample identification number. The second and third lines include a description of the sample content, the date, and the identity of the inoculum.

Since the primary purpose of a sample ID is to provide a link between a physical sample and the computer record that describes the sample, it saves time to rely on Sample IDs that can be scanned by a reader or even a smartphone [27,28] (**Fig 2**). Barcodes are special fonts to print data in a format that can be easily read by an optical sensor [29]. There are also newer alternatives, such as quick response (QR) codes, data matrices, or radio-frequency identification (RFID), to tag samples [30,31]. QR codes and data matrices are 2D barcodes that are cheaper to generate than RFID tags and store more data than traditional barcodes [27]. Nevertheless, these technologies encode a key that points to a database record.

Uniqueness is the most important property of the data encoded in barcodes, and the use of unique and persistent identifiers is a critical component of the Findability of your (meta)data [24]. Several vendors now offer products with 2D barcodes printed on the side or bottom of the tube. It is common for such products, as well as lab-made reagents, to be split across multiple tubes or moved from one tube to another. In these cases, each of these “new” samples should have unique barcodes. A barcoding system can therefore facilitate the accurate identification of “parent” samples (e.g., a stock solution with ID X) and the unique “child” samples derived from them (e.g., aliquots of the stock solution with IDs Y and Z).

### Human-readable IDs

While computer-readable IDs should be the main ID used when tracking a sample or supply, it is sometimes necessary for laboratory personnel to have a secondary sample ID they can read without the use of any equipment or while doing manual operations (i.e., handling samples).

To make an identifier readable by humans, it is best to keep the ID short and use their structure to provide contextual information. For example, the use of a prefix may help interpret the ID. For example, the ID #CHEM1234 would naturally be interpreted as a chemical or #MCUL2147 as a mammalian culture (**Fig 2**).

Since these identifiers do not need to map to a unique database entry, human-readable IDs do not have the same uniqueness requirements as computer-readable IDs. For example, it may be acceptable to allow 2 labs using the same software environment to use the same human-readable ID, because this ID will only be seen in the context of a single lab. The software system should maintain the integrity of the relationships between the human-readable ID and the computer-readable ID by preventing users from editing these identifiers.

## Rule 5: Label everything

Print labels to identify supplies, equipment, samples, storage locations, and any other physical objects used in your lab. Many labs are extensively relying on handwritten markings that create numerous problems [17]. A limited amount of information can be written on small sample containers, and like manually generated sample names, handwritten labels can be difficult to read or interpret.

Some labels are self-contained. For example, shipping labels include all the information necessary to deliver a package. However, in a laboratory environment, a sample label must not only identify a physical sample but also establish a connection to a record describing the sample and the data associated with it (**Fig 2**).

### Content of a label

Only 2 pieces of information are necessary on a label: a computer-readable Sample ID printed as a barcode and a human-readable Sample ID to make it easier for the researcher to work with the sample. If there is enough space to print more information on the label, your research needs should inform your label design. Ensure you have sufficient space to meet regulatory labeling requirements (e.g., biosafety requirements, hazards) and if desired, information such as the sample type, sample creator, date (e.g., of generation or expiration), or information about related samples (e.g., parent/child samples).

### Label printing technology

Putting in place a labeling solution requires the integration of several elements, but once configured, proper use of label printing technologies makes it much faster and easier to print labels than to label tubes manually.

There are many types of label printers on the market today and most are compatible with the Zebra Programming Language (ZPL) standard [32]. Labeling laboratory samples can be challenging due to harsh environmental conditions: exposure to liquid nitrogen or other chemicals, as well as ultra-low or high temperatures, will require specialized labels. For labeling plastic items, thermal transfer will print the most durable labels, especially if used with resin ribbon instead of wax, while inkjet printers can print durable labels for use on paper [33–35]. Furthermore, laboratory samples can be generated in a broad range of sizes, so labels should be adapted to the size of the object they are attached to. A high-resolution printer (300 dpi or greater) will make it possible to print small labels that will be easy to read by humans and scanners. Finally, select permanent or removable labels based on the application. Reusable items should be labeled with removable labels, whereas single-use containers are best labeled with permanent labels.

Label printing software applications can take data from a database or a spreadsheet and map different columns to the fields of label templates, helping to standardize your workflows (Rule 8). They also support different formats and barcode standards. Of course, the label printing software needs to be compatible with the label printer. When selecting a barcode scanner, consider whether it supports the barcode standards that will be used in your label, as well as the size and shape of the barcodes it can scan. Inexpensive barcode scanners will have difficulty reading small barcodes printed on curved tubes with limited background, whereas professional scanners with high performance imagers will effectively scan more challenging labels. When used, barcode scanners transmit a unique series of characters to the computer. How these characters are then used depends on the software application in which the barcode is read. Some applications will simply capture the content of the barcode. Other applications will process barcoded data in real-time to retrieve the content of the corresponding records.

## Rule 6: Manage your data proactively

Many funding agencies now require investigators to include a data management and sharing plan with their research proposals [36,37], and journals have data-sharing policies that authors need to uphold [38]. However, the way many authors share their data indicates a poor understanding of data management [39,40]. Data should not be managed only when publishing the results of a project, they should be managed before the data collection starts [41]. Properly managed data will guide project execution by facilitating analysis as data gets collected (**Fig 1**). Projects that do not organize their data will face difficulties during analysis, or worse, a loss of critical information that will negatively impact progress.

### Use databases to organize your data

It can be tempting to only track data files through notebook entries or dump them in a shared drive (more in Rule 9). That simple data management strategy makes it very difficult to query data that may be spread across multiple files or runs, especially because a lot of contextual information must be captured in file names and directory structures using conventions that are difficult to enforce. Today, most data are produced by computer-controlled instruments that export tabular data (i.e., rows and columns) that can easily be imported into relational databases. Data stored in relational databases (e.g., MySQL) are typically explored using standard query language (SQL) and can be easily analyzed using a variety of statistical methods (**Table 1**). There are also no-code and low-code options, such as the Open Science Framework (https://osf.io/) [42], AirTable, and ClickUp, which can also be used to track lab processes, develop standardized workflows, manage teams, etc.

**Table 1:** Comparison of data management frameworks.

	Relational Databases	NoSQL Databases	Data Lakes	Data Warehouses
Primary Application	Querying and analyzing structured data organized in tables (i.e., columns and rows)	Storing and querying unstructured and semi-structured data; type depends on data model	Storing raw/pre-processed structured and unstructured data	Storing and analyzing large-scale structured data (often SQL-like)
Advantages	• Widely used • Consistent structure • Standardized structured query language (SQL)	• Data schema is flexible • Complementary to relational databases	• Simple data ingestion • Facilitates analysis of heterogenous data	• Higher storage capabilities • Optimized for analysis
Limitations	• Scalability (performance decreases as data volume increases) • Data schema is fixed	• Often optimized for certain use cases • No standard query language • Analysis capabilities depend on technology	• Metadata management is critical for data access and interrogation	• Data must be processed for ingestion • Typically process-focused (potential for lower scope)
Example technologies	• MySQL • Oracle • Microsoft SQL server • IBM Db2 • PostgreSQL • Amazon Relational Database Service (RDS)	Document-oriented: • MongoDB Key-value pair: • DynamoDB Column-oriented: • Apache Cassandra • Google Bigtable Graph-based: • Neo4j	• Amazon S3 • Microsoft Azure Data Lake • Apache Hadoop • Google Cloud Storage • *Databricks	• *Snowflake Data Cloud • Amazon Redshift • Google BigQuery • Apache Hive • IBM Db2 Warehouse

*These technologies have been dubbed “lakehouses” that integrate components of data lakes and data warehouses.

In the age of big data applications enabled by cloud computing infrastructures, there are more ways than ever to organize data. Today, NoSQL (not only SQL) databases [43–45], data lakes [46–48], and data warehouses [49,50] provide additional avenues to manage complex sets of data that may be difficult to manage in relational databases (**Table 1**). All these data management frameworks make it possible to query and analyze data, depending on the size, type, and structure of your data as well as your analysis goals. NoSQL databases can be used to store and query data that is unstructured or otherwise not compatible with relational databases. Different NoSQL databases implement different data models to choose from depending on your needs (**Table 1**). Data lakes are primarily used for storing large-scale data with any structure. It is easy to input data into a data lake, but metadata management is critical for organizing, accessing, and interrogating the data. Data warehouses are best suited for storing and analyzing large-scale structured data. They are often SQL-like and are sometimes optimized for specific analytical workflows. These technologies are constantly evolving and the overlap between them is growing as captured in the idea of “lakehouses” such as Databricks and Snowflake Data Cloud (https://www.snowflake.com/en/) (**Table 1**).

When choosing a data management system, labs must consider the trade-off between the cost of the service and the accessibility of the data (i.e., storage in a data lake may be cheaper than in a data warehouse, but retrieving/accessing the data may be more time-consuming or costly) [51]. Many companies offer application programming interfaces (API) to connect their instruments and/or software to databases. In addition, new domain-specific databases continue to be developed [52]. If necessary, it is also possible to develop your own databases for particular instruments or file types [53]. Nevertheless, when uploading your data to a database, it is recommended to import them as interoperable nonproprietary file types (e.g., .csv instead of .xls for tabular data; .gb (GenBank flat file https://www.ncbi.nlm.nih.gov/genbank/) instead of .clc (Qiagen CLC Sequence Viewer format [54]) for gene annotation data; see Rule 4 of [51] for more), so that the data can be accessed if a software is unavailable for any reason and to facilitate date sharing using tools such as git (Rule 10) [14,24].

### Link data to protocols

One of the benefits of data organization is the possibility of capturing critical metadata describing how the data were produced. Many labs have spent years refining protocols to be used in different experiments. Many of these protocols have minor variations that can significantly alter the outcome of an experiment. If not properly organized, this can cause major reproducibility issues and can be another uncontrolled source of technical variation. By linking protocol versions to the associated data that they produced (ideally all the samples generated throughout the experiment), it is possible to use this metadata to inform data reproducibility and analysis efforts.

### Capture context in notebook entries

Organizing data in databases and capturing essential metadata describing the data production process can greatly simplify the process of documenting research projects in laboratory notebooks [55]. Instead of needing to include copies of the protocols and the raw data produced by the experiment, the notebook entry can focus on the context, purpose, and results of the experiment. In the case of electronic lab notebooks (ELNs; e.g., SciNote, LabArchives, and eLabJournal), entries can benefit from providing links to previous notebook entries, the experimental and analytical protocols used, and the datasets produced by the workflows. ELNs also bring additional benefits like portability, standardized templates, and improved reproducibility. Finally, notebook entries should include the interpretation of the data as well as a conclusion pointing to the next experiment. The presence of this rich metadata and detailed provenance is critical to ensuring the FAIR principles are being met and your experiments are reproducible [24].

## Rule 7: Separate parameters and variables

Not all the data associated with an experiment are the same. Some data are controlled by the operator (i.e., parameters), whereas other data are observed and measured (i.e., variables). It is necessary to establish a clear distinction between set parameters and observed variables to improve reproducibility and analysis.

When parameters are not clearly identified, lab personnel may be tempted to change parameter values every time they perform experiments, which will increase the variability of observations. If, instead, parameter values are clearly identified and defined, then the variance of the observations produced by this set of parameters should be smaller than the variance of the observations produced using different parameter values.

Separating and recording the parameters and variables associated with an experiment makes it possible to build statistical models that compare the observed variables associated with different parameter values [41,56]. It also enables researchers to identify and account for both the underlying biological factors of interest (e.g., strain, treatment) and the technical and random (noise) sources of variation (e.g., batch effects) in an experiment [56].

Utilizing metadata files is a convenient way of reducing variability caused by parameter value changes. A metadata file should include all the parameters needed to perform the same experiment with the same equipment. In an experimental workflow, pairing a metadata file with the quantified dataset is fundamental to reproducing the same experiment later [51,55,57]. Additionally, metadata files allow the user to assess whether multiple experiments were performed using the same parameters.

## Rule 8: Standardize your workflows

### Track your parameters from beginning to end

Experimental parameters have a direct influence on observations. However, some factors may have indirect effects on observations or affect observations further downstream in a pipeline. For example, the parameters of a DNA purification process may indirectly influence the quality of sequencing data derived from the extracted DNA.

To uncover such indirect effects, it is necessary to capture the sequence of operations in workflows. For the above example, this would include the DNA extraction, preparation of the sequencing library, and the sequencing run itself. When dealing with such workflows, it is not possible to use a single Sample ID as the key connecting different datasets as in Rule 4. The workflow involves multiple samples (i.e., the biological sample or tissue, the extracted DNA, the sequencing library) that each have their own identifier. Comprehensive inventory and data management systems will allow you to track the sample lineage and flows of data produced at different stages of an experimental process.

Recording experimental parameters and workflows is especially critical when performing new experiments, since they are likely to change over time. As they are finalized, this information can be used to develop both standardized templates for documenting your workflow, as well as metrics for defining the success of each experiment, which can help you to optimize your experimental design and data collection efforts (**Fig 1**).

### Document your data processing pipeline

After the experimental data are collected, it is important to document the different steps used to process and analyze the data, such as if normalization was applied to the data, or if extreme values were not considered in the analyses. The use of ELNs and LIMS can facilitate standardized documentation: creating templates for experimental and analysis protocols can ensure that all the necessary information is collected, thereby improving reproducibility and publication efforts [55,58].

Similarly, thorough source-code documentation is necessary to disseminate your data and ensure that other groups can reproduce your analyses. There are many resources on good coding and software engineering practices [14–16,59], so we only touch on a few important points. Developing a “computational narrative” by writing comments alongside your code or using interfaces that allow for markdown (e.g., Jupyter notebooks, R Markdown) can make code more understandable [60–62]. Additionally, using syntax conventions and giving meaningful names to your code variables increases readability (i.e., use average_mass = 10 instead of am = 10). Furthermore, documenting the libraries or software used and their versions is necessary to achieve reproducibility. Finally, implementing a version control system, such as git, protects the provenance of your work and enables collaboration [63].

## Rule 9: Avoid data silos

Depending on your workflows, you may collect information from different instruments or use several databases to store and interact with different types of data. Care must be taken to prevent any of these databases from becoming data silos: segregated groups of data that restrict collaboration and make it difficult to capture insights resulting from data obtained by multiple instruments [47,49,64]. Data lakes and data warehouses are good solutions for integrating data silos [47,49,64].

Data silos not only stymie research efforts but also raise significant security issues when the silo is the sole storage location. Keeping your data management plan up-to-date with your current needs and utilizing the right databases for your needs can prevent this issue (Rule 6). Regardless, it is crucial to back up your data in multiple places for when a file is corrupted, a service is unavailable, etc. Optimally, your data should always be saved in 3 different locations: 2 on-site and 1 in the cloud [51]. Of course, precautions should always be taken to ensure the privacy and security of your data online and in the cloud [65,66].

### Never delete data

As projects develop and data accumulates, it may be tempting to delete data that no longer seems relevant. Data may also be lost as computers are replaced, research personnel leave, and storage limits are reached. Poorly managed data can be easily lost simply because it is unorganized and difficult to query. However, while data collection remains expensive, data storage continues to get cheaper, so there is little excuse for losing or deleting data today. The exception may be intermediary data that is generated by reproducible data processing pipelines, which can be easily regenerated if and when necessary. Most data files can also be compressed to combat limitations on storage capacity.

Properly organized data is a treasure trove of information waiting to be discovered. By using computer-generated sample IDs (Rule 4) and data lakes/warehouses (Rule 6) to link data collected on different instruments, it is possible to extract and synthesize more information than originally intended in the project design. Data produced by different projects using common workflows (Rule 8) can be analyzed to improve workflow performance. Data from failed experiments can be used to troubleshoot a problem affecting multiple projects.

## Rule 10: Place your lab in context

Once you have developed a common culture (Rule 1), inventoried your laboratory (Rules 2 and 3), labeled your samples and equipment with computer-generated IDs (Rules 4 and 5), standardized your parameters and workflows (Rules 7 and 8), and backed up your data in several databases (Rules 6 and 9), what comes next?

### Track processes occurring outside the lab

Laboratory operations and the data they produce are increasingly dependent on operations that take place outside of the lab. For example, the results of a PCR assay will be affected by the primer design algorithm and the values of its parameters. They will also be affected by the quality of the primers manufactured by a specialized service provider. Even though the primer design and primer synthesis are not taking place in the lab, they are an integral part of the process of generating PCR data. They should therefore be captured in data flows (Rule 8). Furthermore, the software and computational steps used to design experiments and analyze data they produce must also be properly recorded, to identify as many factors that may affect the data produced in the lab as possible.

### Increase the accessibility of your work

There are several ways to place your lab in the greater scientific context and increase reproducibility. As discussed, using standardized, non-proprietary file types can increase ease of access within a lab and across groups [14,51]. You may also choose to make your data and source code public in an online repository to comply with journal requirements, increase transparency, or allow access to your data by other groups [67]. In addition, data exchange standards, such as the Synthetic Biology Open Language [68,69], increase the accessibility and reproducibility of your work.

### Practice makes perfect

Whereas traditional data management methods can restrict your analyses to limited subsets of data, centralized information management systems (encompassing relational and NoSQL databases, metadata, sample tracking, etc.) facilitate the analysis of previously disparate datasets. Given the increasing availability and decreasing cost of information management systems, it is now possible for labs to produce, document, and track a seemingly endless amount of samples and data, and use these to inform their research directions in previously impossible ways. When establishing your LIMS, or incorporating new experiments, it is better to capture more data than less. As you standardize your workflows (Rule 8), you should be able to establish clear metrics defining the success of an experiment and to scale the amount of the data you collect as needed.

While there are plenty of existing ELN and LIMS services to choose from (see Rule 6 and Rule 2, respectively), none are a turnkey solution (S1 Supporting information). All data management systems require configuration and optimization for an individual lab. Each service has its own benefits and limitations your group must weigh. Coupled with the need to store your data with multiple backup options, thoughtful management practices are necessary to make any of these technologies work for your lab. The 10 rules discussed here should provide both a starting place and continued resource in the development of your lab information management system. Remember that developing a LIMS is not a one-time event; all lab members must contribute to the maintenance of the LIMS and document their supplies, samples, and experiments in a timely manner. Although it might be an overwhelming process to begin with, careful data management will quickly benefit the data, users, and lab through saved time, standardized practices, and more powerful insights [17,18].

## Conclusions

Imparting a strong organizational structure for your lab information can ultimately save you both time and money if properly maintained. We present these 10 rules to help you build a strong foundation in managing your lab information so that you may avoid the costly and frustrating mistakes we have made over the years. By implementing these 10 rules, you should see some immediate benefits of your newfound structure, perhaps in the form of extra fridge space or fewer delays waiting for a reagent you did not realize was exhausted. In time, you will gain deep insights into your workflows and more easily analyze and report your data. The goal of these rules is also to spur conversation about lab management systems both between and within labs as there is no one-size-fits-all solution for lab management. While these rules provide a great starting point, the topic of how to manage lab information is something that must be a constant dialogue. The lab needs to discuss what is working and what is not working to assess and adjust the system to meet the needs of the lab. This dialogue must also be extended to all new members of the lab as many of these organizational steps may not be intuitive. It is critical to train new members extensively and to ensure that they are integrated into the lab’s common culture or else you risk falling back into bad practices. If properly trained, lab members will propagate and benefit from the organizational structure of the lab.
